# Truncated Transfer Matrix-Based Regularization for Impact Force Localization and Reconstruction

**DOI:** 10.3390/s25185712

**Published:** 2025-09-12

**Authors:** Bing Zhang, Xinqun Zhu, Jianchun Li

**Affiliations:** School of Civil and Environmental Engineering, University of Technology Sydney, Ultimo, NSW 2007, Australia; bing.zhang-5@alumni.uts.edu.au (B.Z.); jianchun.li@uts.edu.au (J.L.)

**Keywords:** impact force identification, structural health monitoring, inverse problem, truncated transfer matrix, sparse regularization, modal superposition, numerical and experimental validation

## Abstract

Civil infrastructure, such as bridges and buildings, is susceptible to damage from unforeseen low-speed impacts during service. Impact force identification from dynamic response measurements is essential for structural health monitoring and structural design. Force identification is an ill-posed inverse problem, and the regularization technique is widely used to solve this problem using a full transfer matrix. However, existing regularization techniques are not suitable for large-scale practical structures due to the high computational cost for the inverse calculation of a high-dimensional transfer matrix, and impact excitation locations are often unknown in practice. To address these challenges, a novel two-step truncated transfer matrix-based impact force identification method is proposed in this study. In the first step, a sparse regularization-based technique is developed to determine unknown force locations using modal superposition. In the second step, the full transfer matrix is truncated by time windows corresponding to short durations of impact excitations, and a Tikhonov regularization-based technique is adopted to reconstruct the time history of impact forces. The proposed method is verified numerically on a simply supported beam and experimentally on a 10 m steel–concrete composite bridge deck. The results show that the proposed method could determine the impact locations and reconstruct the time history of impact forces accurately. Compared with existing Tikhonov and sparse regularization methods, the proposed method demonstrates superior accuracy and computational efficiency for impact force identification. The robustness of the proposed method to noise level and the number of modes and sensors is investigated. Experimental studies for both single-force and multiple-force localization and identification are conducted. The results indicate that the proposed method is efficient and accurate in identifying impact forces.

## 1. Introduction

Civil infrastructures are often subject to damage caused by low-velocity impacts [1], especially for transportation infrastructure. Bridges are essential components of transportation infrastructure, and they are susceptible to unforeseen low-speed impacts during service, such as vehicle collisions [2,3] or ship collisions [4], the impact of ocean wave forces [5], and rockfall impacts [6]. The accurate identification of dynamic loads acting on civil structures is crucial in engineering practice for structural strength analysis, reliability analysis, structural health monitoring, and condition assessment [7,8,9]. However, directly measuring these impact forces is often infeasible due to the inaccessibility of impact locations. Indirect force identification is often used to determine the external forces from measured dynamic responses [10]. Different methods have been developed to identify impact forces on structures. LeCleric et al. [11] proposed a neural network approach to the impact detection of an aircraft composite panel. Park et al. [12] identified impacts on a complex structure with built-in sensors using transfer functions. Force identification is a typical ill-posed inverse problem, which is very sensitive to measurement noise [13]. Different stabilization techniques have been used to enhance the robustness of impact force identification against measurement noise, as listed in one literature review [14]. For large-scale practical structures, it is still a big challenge to identify multiple impact forces without prior impact location information.

Regularization methods are commonly used for force identification problems, such as the Tikhonov and sparse regularization methods. Tikhonov regularization is normally based on *l*_2_-norm. Zhu et al. [15] used Tikhonov regularization to identify moving loads on a continuous beam based on the measured structural vibration response. Jacquelin et al. [16] compared the performance of Tikhonov regularization and truncated singular value decomposition (TSVD) for reconstructing the time history of impact force acting on an aluminum plate in the time domain. Kalhori et al. [17] applied the Tikhonov regularization method to reconstruct the time history and localize the impact force acting on a composite panel. Jia et al. [18] proposed a weighted Tikhonov regularization method for identifying random dynamic force in the frequency domain, where the weighting matrix depends on the frequency response function. Jayalakshmi et al. [19] presented a modified Tikhonov method to reconstruct dynamic force from multiple sensors, which was numerically verified on a shear building. Pourzeynali et al. [20] developed a moving load identification method based on the explicit form of the Newmark-β method and Tikhonov regularization. As noted above, these *l*_2_-norm regularization methods tend to produce over-smooth solutions and fail to capture the sharp impulse of impact forces [21,22]. The accuracy of reconstructed impact forces might be insufficient for high-damping system designs [23], which are sensitive to external excitations.

Sparse regularization has received significant attention in dynamic force identification. *l*_1_-norm regularization is commonly employed as the standard sparse regularization technique. Within the Bayesian framework, Samagassi et al. [24] applied a relevance vector machine approach based on the Daubechies wavelet and *l*_1_-norm penalty for reconstructing multiple impact forces acting on a beam. Qiao et al. [25] proposed an enhanced sparse regularization method for impact force identification based on weighted *l*_1_-norm minimization. To consider the intrinsic structure of the impact force where nonzero elements occur in groups, a sparse group regularization method minimizing the mixed *l*_2,1_-norm has been proposed for impact force identification [26]. Liu et al. [27] developed a novel sparse regularization method with a generalized minimax-concave (GMC) penalty to address the impact force identification problem. Combining a redundant dictionary, sparse regularization can also be applied to moving load identification. Zhong et al. [28] used a sparse regularization approach for traffic load monitoring based on an analytical model and redundant dictionary. Based on the redundant concatenated dictionary and weighted *l*_1_-norm regularization method, Pan et al. [29] developed a hybrid method for moving force identification. Additionally, several nonconvex sparse regularization methods [30,31] have been proposed to recover the time history of impact forces for sparser and more accurate solutions. From the above, various methods, such as mode superposition, state space functions, and unit impulse response functions, are employed to construct transfer matrices. The constructed transfer matrix from these methods is a lower triangular Toeplitz matrix, with strong correlations among the adjacent columns in this matrix. As the impact force has several nonzero elements in the excitation time interval, it is challenging to identify the force using sparse regularization.

For all the above regularization methods, the impact force locations are assumed as pre-known. In practice, the impact force locations are often unknown, and this poses additional challenges during the force identification process. Wang et al. [32] identified the location and amplitude of an unknown impact force acting on a simply supported beam in the time and frequency domains. Li et al. [33] proposed a method for impact localization and identification. The location was first determined with an error functional indicator using the complex method. The identification of impact time history was then considered a constrained optimization problem. Wambacq et al. [34] presented an algorithm to localize and identify forces in the frequency domain. Leveraging the group sparse feature, Feng et al. [35] utilized the external force group sparse feature and developed an original time domain group sparsity regularization method, named group relevance vector machine, to localize and reconstruct external forces on structures based on structure responses only. Liu et al. [36] also used the force vector group sparse feature and proposed a novel impact force identification method based on nonconvex overlapping group sparsity (NOGS), allowing for the simultaneous localization and time history recovery of impacts from limited measurements. Zhang et al. [37] proposed a generalized transmissibility-based method to localize and reconstruct the impact force in modal coordinates via a single sensor. Xiao et al. [38] presented an adaptive wavelet regularization time domain deconvolution method for impact force identification, and the adaptive impact window covering the entire impact duration was obtained to reduce signal length for deconvolution. Li et al. [39] introduced a dynamic reduced dictionary to improve computational efficiency.

As shown above, the construction of the transfer matrix significantly influences the force identification results. Especially for large-scale structures in practice, the transfer matrix is a high-dimensional matrix, and the computational cost for inverse calculation is high. Also, the ill-posedness of inverse problems significantly affects the accuracy of force identification. This paper proposes a truncated transfer matrix-based regularization method for impact force identification and a sparse regularization method for force localization. By eliminating the effect of unloading interval, the transfer matrix could be truncated to the short time interval of impact force, and the accuracy and computational efficiency of the force identification can be increased. Further, based on the modal parameters of structures and sparse feature of impact force, the force location can be determined. Numerical and experimental studies have been conducted to verify the performance of the proposed method.

## 2. Theory

### 2.1. The Dynamics of a Simply Supported Beam Under Impact Forces

A simply supported Euler–Bernoulli beam is subjected to an impact force with an unknown excitation location. The beam is assumed to have a constant cross-section with uniform mass per unit length and linear, viscous proportional damping. The effects of shear deformation and rotary inertia are neglected. The dynamic governing equation is given as follows:

(1) $$M\ddot{x(t)}+C\dot{x(t)}+Kx(t)=LF(t)$$

where M, C, and K  are mass, damping, and stiffness matrices, respectively. $\ddot{x}\left(t\right)$, $\dot{x}(t)$, and x(t) are the acceleration, velocity, and displacement response vectors of the beam, respectively. F(t) is the input force vector, and L is the mapping matrix for the external input forces. Based on modal superposition, the dynamic response $\ddot{x}(t)$ can be described as follows:

(2) $$\ddot{x}\left(t\right)=\sum_{r=1}^{N}\Phi_{r}{\ddot{q}}_{r}\left(t\right)\;$$

where $\Phi_{r},{\ddot{q}}_{r}(t)$ are the $r^{th}$ mode shape and modal coordinate, respectively. N is the number of modes.

Substituting Equation (2) into Equation (1), based on orthogonality, the following can be obtained:

(3) $$M_{r}\ddot{q_{r}}\left(t\right)+C_{r}\dot{q_{r}}\left(t\right)+K_{r}q_{r}\left(t\right)=f_{r}\left(t\right),\;(r=1,\ldots ,N)$$

where ${\;M}_{r}={\Phi_{r}}^{T}M\Phi_{r},C_{r}={\Phi_{r}}^{T}C\Phi_{r},K_{r}={\Phi_{r}}^{T}K\Phi_{r},andf_{r}\left(t\right)={\Phi_{r}}^{T}LF(t)$ are the modal mass, modal damping, modal stiffness, and modal force, respectively.

Equation (3) is expressed as follows:

(4) $$\ddot{q_{r}}\left(t\right)+{{2\xi }_{r}\omega }_{r}\dot{q_{r}}\left(t\right)+{\omega_{r}}^{2}q_{r}\left(t\right)=f_{r}\left(t\right)/M_{r},\;(r=1,\ldots ,N)$$

where ${\omega_{r}}^{2}=K_{r}/M_{r}$,$\;\xi_{r}=C_{r}/2\omega_{r}M_{r}$ denotes the damping ratio for the $r^{th}$ mode. The modal acceleration response can be obtained from Equation (4) as follows:

(5) $${\ddot{q}}_{r}\left(t\right)=\int_{0}^{t}{\ddot{h}}_{r}(t-\tau )f_{r}\left(\tau \right)d\tau$$

with

(6) $${\ddot{h}}_{r}\left(t\right)=\frac{1}{M_{r}\omega_{dr}}e^{-\xi_{r}\omega_{r}t}[\left({\xi_{r}}^{2}{\omega_{r}}^{2}-{\omega_{dr}}^{2}\right)\sin\omega_{dr}t-{2\xi_{r}\omega_{dr}\omega }_{r}cos\omega_{dr}t]$$

where $\omega_{dr}$ is the $r^{th}$ inherent circular frequency of the undamped system, and $\omega_{dr}=\omega_{r}\sqrt{1-{\xi_{r}}^{2}}$ is the $r^{th}$ actual vibration circle frequency of the system with the damping $C_{r}$.

From Equations (2) and (5), the acceleration response can be obtained as

(7) $$\ddot{x}\left(t\right)=\int_{0}^{t}R\left(t-\tau \right)F(t)d\tau$$

where R is the impulse response function matrix

(8) $$R\left(t-\tau \right)=\sum_{r=1}^{n}\frac{\Phi_{r}{\Phi_{r}}^{T}L}{M_{r}\omega_{dr}}e^{-\xi_{r}\omega_{r}(t-\tau )}[\left({\xi_{r}}^{2}{\omega_{r}}^{2}-{\omega_{dr}}^{2}\right)\sin\omega_{dr}(t-\tau )-{2\xi_{r}\omega_{dr}\omega }_{r}cos\omega_{dr}(t-\tau )]$$

Equation (7) can be discretized as the matrix convolution form in the time duration from $t_{1}$ to $t_{nt}$ as follows:

(9) $$\left\{\begin{array}{c} \begin{array}{c} \ddot{x}(t_{1})\\ \ddot{x}(t_{2}) \end{array}\\ \vdots \\ \ddot{x}(t_{nt}) \end{array}\right\}=∆t\left[\begin{array}{ccc} \begin{array}{c} R\left(t_{1}\right)\\ R\left(t_{2}\right) \end{array} & \begin{array}{c} 0\\ R\left(t_{1}\right) \end{array} & \begin{array}{c} \begin{array}{cc} \ldots & 0 \end{array}\\ \begin{array}{cc} \ldots & 0 \end{array} \end{array}\\ \vdots & \vdots & \begin{array}{cc} \ddots & \vdots \end{array}\\ R\left(t_{nt}\right) & R\left(t_{nt-1}\right) & \begin{array}{cc} \ldots & R\left(t_{1}\right) \end{array} \end{array}\right]\left\{\begin{array}{c} \begin{array}{c} F(t_{1})\\ F(t_{2}) \end{array}\\ \vdots \\ F(t_{nt}) \end{array}\right\}$$

where nt is the number of time steps. ∆t is the time interval determined by sampling frequency. Equation (9) can be simplified into a matrix–vector form as follows:

(10) $$A_{(ns\times nt)\times 1}=H_{\left(ns\times nt)\times (nl\times nt\right)}F_{(nl\times nt)\times 1}$$

where $A_{(ns\times nt)\times 1}$ is the accelerations response vector. ns is the number of measurement points. $F_{(nl\times nt)\times 1}$ is the force vector to be identified. nl is the number of load locations, and the transfer matrix $H_{\left(ns\times nt)\times (nl\times nt\right)}$ is a lower triangular Toeplitz matrix.

Considering the measurement noise, the following can be expressed:

(11) A=HF+e

where the vector e represents the inevitable measurement error that corrupts the actual response A.

### 2.2. Force Localization

In practice, load locations may be unknown. Force location information has sparse features in the modal force. This inherent sparsity reflects the fact that impacts occur at isolated points rather than across the entire structure. When the impact forces occur at discrete locations on the structure, only their corresponding columns of the mapping matrix contribute to the modal force. This means only modal force vectors at the impact locations are nonzero entries, and the vectors at other locations are approximately zero. Based on this information, a new method using singular value decomposition (SVD) and sparse regularization is proposed to localize the forces. Equation (5) can be discretized as below:

(12) $$\left[\begin{array}{c} {\ddot{q}}_{r}\left(t_{1}\right)\\ {\ddot{q}}_{r}\left(t_{2}\right)\\ \begin{array}{c} \vdots \\ {\ddot{q}}_{r}\left(t_{nt-1}\right)\\ {\ddot{q}}_{r}\left(t_{nt}\right) \end{array} \end{array}\right]=∆t\left[\begin{array}{ccc} \begin{array}{cc} {\ddot{h}}_{r}\left(t_{1}\right) & 0\\ {\ddot{h}}_{r}\left(t_{2}\right) & {\ddot{h}}_{r}\left(t_{1}\right) \end{array} & \begin{array}{c} \ldots \\ \ldots \end{array} & \begin{array}{cc} 0 & 0\\ 0 & 0 \end{array}\\ \begin{array}{cc} \vdots & \vdots \end{array} & \ldots & \begin{array}{cc} \vdots & \vdots \end{array}\\ \begin{array}{cc} {\ddot{h}}_{r}\left(t_{nt-1}\right) & {\ddot{h}}_{r}\left(t_{nt-2}\right)\\ {\ddot{h}}_{r}\left(t_{nt}\right) & {\ddot{h}}_{r}\left(t_{nt-1}\right) \end{array} & \begin{array}{c} \ldots \\ \ldots \end{array} & \begin{array}{cc} {\ddot{h}}_{r}\left(t_{1}\right) & 0\\ {\ddot{h}}_{r}\left(t_{2}\right) & {\ddot{h}}_{r}\left(t_{1}\right) \end{array} \end{array}\right]\left[\begin{array}{c} f_{r}\left(t_{1}\right)\\ f_{r}\left(t_{2}\right)\\ \begin{array}{c} \vdots \\ f_{r}\left(t_{nt-1}\right)\\ f_{r}\left(t_{nt}\right) \end{array} \end{array}\right]$$

where nt is the number of time steps. ∆t is the time interval determined by sampling frequency. Equation (12) can be written in a matrix–vector form as

(13) $${\ddot{q}}_{r}={\ddot{H}}_{r}f_{r},\;(r=1,\ldots ,n)$$

where ${\ddot{q}}_{r}$ is the $r^{th}$ mode amplitude vector. $f_{r}$ is the $r^{th}$ modal force vector to be identified, and the $r^{th}$ transfer matrix ${\ddot{H}}_{r}\in R^{nt\times nt}$ is a lower triangular Toeplitz matrix. ${\ddot{q}}_{r}$ is extracted from the response by modal decomposition.

Based on the mode superposition method, the modal force can be derived as

(14) $$f(t)=\Phi^{T}LF(t)$$

where $f(t)={\left[f_{1}(t)\;f_{2}(t)\ldots f_{n}(t)\right]}^{T}$ is the modal force vector. $f_{r}(t)$ is the rth modal force. $L=[L_{1}L_{2}\ldots L_{nl}]$ is the mapping matrix. $L_{i}={\left[0\ldots 0\;1\;0\ldots 0\right]}^{T}$ is the ith force mapping vector, and the element value 1 represents the *i*th force location.

Equation (14) can be discretized as

(15) $$f=\sum_{i=1}^{nl}\Phi^{T}L_{i}F_{i}$$

where $f={\left[f_{1}\;f_{2}\ldots f_{n}\right]}^{T}$ is the modal force matrix, and $f_{r}$ is the *r*th modal force vector.

Assuming the sparse feature of impacts and that each impact is not simultaneous and overlapped, the modal force matrix can be split as

(16) $$f=[f_{1}^{\prime }f_{2}^{\prime }\ldots f_{nl}^{\prime }]$$

where $f_{i}^{\prime }$ is the matrix that contains the main part of the *i*th impact force as follows:

(17) $$f_{i}^{\prime }={\Phi^{T}L}_{i}F_{i}^{\prime },\;(i=1,\ldots ,nl)$$

where $F_{i}^{\prime }$ is the main part of the *i*th impact force vector.

To characterize the sources, an *nt*-by-*n* matrix of *n* modal forces is assembled over *nt* spectral lines (with *n* ≤ *nt*) and decomposed via SVD as

(18) $$f_{i}^{\prime }=U_{i}\Sigma_{i}{V_{i}}^{T},\;(i=1,\ldots ,nl)$$

where $U_{i}=[U_{i,1},U_{i,2},\ldots ,U_{i,m}]$ represents the right singular vectors; ${V_{i}}^{T}=[U_{i,1},U_{i,2},\ldots ,U_{i,m}]$ represents the left singular vectors; $\Sigma_{i}=diag[\sigma_{i,1},\sigma_{i,2},\ldots ,\sigma_{i,m}]$ represents the singular values.

In Equation (17), $f_{i}^{\prime }$ contains only one feature vector of $F_{i}^{\prime }$**.** Equation (18) can be approximated by the first singular value and associated basis vectors as

(19) $$f_{i}^{\prime }\approx U_{i,1}\sigma_{i,1}{V_{i,1}}^{T}$$

Combining Equations (18) and (19), the singular vector $U_{i,1}$ is associated with the modal coefficients and can be expressed as

(20) $$U_{i,1}=\Phi^{T}a_{i}+e,\;(i=1,\ldots ,nl)$$

where $a_{i}=L_{i}a_{i}$, $a_{i}$ is a coefficient. e is the inevitable error. Here ${\{a}_{i,}i=1,2,\ldots nl\}$ are related to the locations of the impact forces, and they can be determined by sparse regularization as

(21) $$\underset{a_{i}}{minmize}\;\;\;{\left\|\Phi^{T}a_{i}-U_{i,1}\right\|}_{2}^{2}+\lambda {\left\|a_{i}\right\|}_{1},\;(i=1,\ldots ,nl)$$

In Equation (21), $U_{i,1}$ is the first right singular vector of the matrix $f_{i}^{\prime }$, which corresponds to the dominant pattern of the measured modal forces at candidate location i. The regularization parameter λ>0 balances data fidelity and sparsity. Minimizing this objective function in Equation (21) yields each sparse coefficient vector $a_{i}$ in which only the element corresponding to the true impact location remains nonzero. Once the force locations are determined, the transfer matrix H can be constructed to identify the force time history associated with all modes.

### 2.3. Truncated Transfer Matrix-Based Regularization Method for Impact Force Identification

In this section, two typical regularization techniques for force identification are introduced first, and then a novel regularization method is proposed for impact force identification.

#### 2.3.1. Tikhonov Regularization Method

Tikhonov regularization, based on minimizing *l*_2_-norm, is a typical method for solving linear inverse problems [15]. Impact force identification, defining a trade-off between the residual and regularized norms, is formulated as

(22) $$\underset{F}{minmize}\;\;\;{\left\|HF-A\right\|}_{2}^{2}+\lambda {\left\|F\right\|}_{2}^{2}$$

where λ>0 is the regularization parameter. The *l*_2_-norm of the impact force ${\left\|F\right\|}_{2}^{2}$ is the regularization term or the penalty term. Here, the ill-posed problem of Equation (11) is improved by introducing an additional term in Equation (22), rendering the problem less sensitive to perturbations. Due to the convexity of Equation (22), Tikhonov regularization always yields an analytic solution with any fixed λ

(23) $$F={\left(H^{T}H+\lambda I\right)}^{-1}H^{T}A$$

As above, the Tikhonov solution is a smooth function of λ, varying over the interval (0,∞).

#### 2.3.2. *l*_1_-Norm Regularization Method for Impact Force Identification

Lasso regression expects many coefficients to be close to zero, with only a small subset to be nonzero. The lasso estimator uses the *l*_1_-penalized least squares criterion to obtain a sparse solution as the following optimization problem [25]:

(24) $$\underset{F}{minmize}\;\;\;{\left\|HF-A\right\|}_{2}^{2}+\lambda {\left\|F\right\|}_{1}$$

where ${\left\|F\right\|}_{1}$ is the *l*_1_-norm of the impact force, which incorporates the sparsity on the unknown force.

#### 2.3.3. Truncated Transfer Matrix-Based Regularization Method

**Figure 1:**
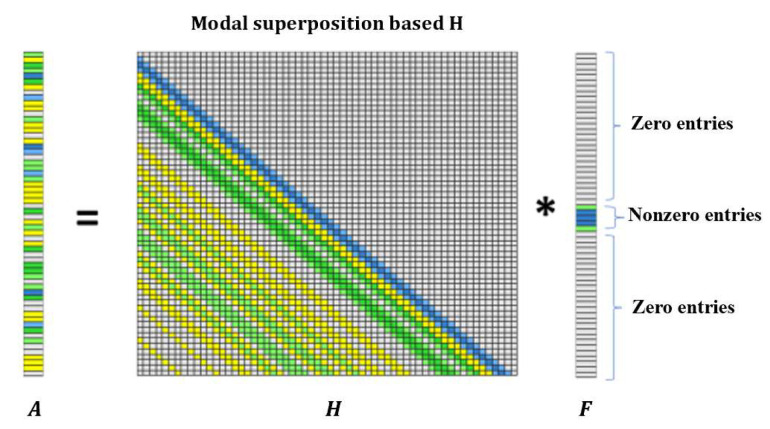
Discrete form of impact force identification (Note: ‘*’ is a mathematical convolution operation).

Previous studies have assumed that the impact force manifests as a triangular pulse with only one nonzero value. Under this simplified assumption, the identified results based on *l*_1_-norm regularization perform well. However, in practical applications, the impact force often comprises multiple nonzero values within specific time intervals. As shown in Figure 1, the impact force could be separated into zero and nonzero entries. Since the transfer matrix is a lower triangular Toeplitz matrix, strong correlations exist among the adjacent columns in this kind of matrix. Consequently, the application of an *l*_1_-norm penalty treats all variables differently and encourages sparsity in individual coefficients. The presence of strong correlations among variables may lead to the elimination of coefficients.

The *l*_1_-norm regularization method, while effective in certain scenarios, exhibits reduced robustness in cases of high correlation, making it less suitable for impact force identification with multiple nonzero values. Conversely, the *l*_2_-norm penalty treats all variables equally and does not encourage sparsity, leading to the potential weakening of impact force values in the presence of noise. To address these limitations, the group lasso regularization method combines the *l*_1_-norm penalty and *l*_2_-norm penalty to achieve improved identification results, as follows:

(25) $$\underset{F}{minmize}\;\;\;{\left\|HF-A\right\|}_{2}^{2}+\lambda \sum_{i}^{GN}{\left\|F_{gi}\right\|}_{2}^{2}$$

where $F_{gi}$ is the force vector at the location i, and *GN* is the number of impact forces applied asynchronously at different locations.

**Figure 2:**
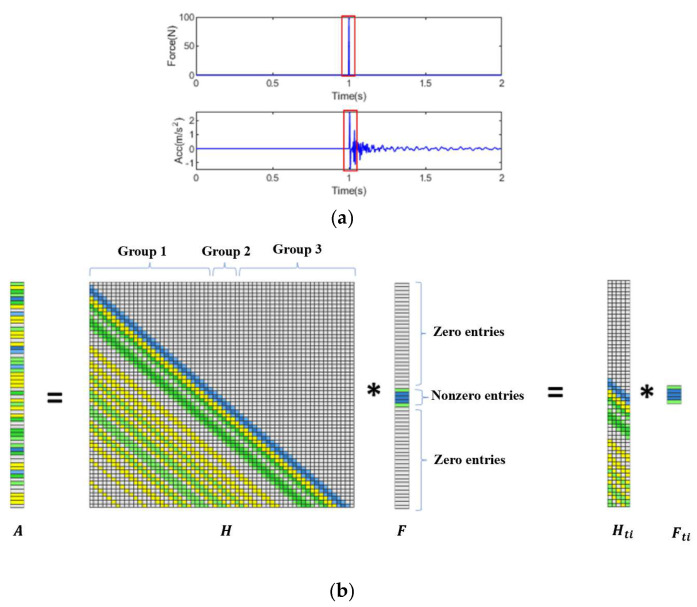
Impact force and response and its truncated transfer matrix. (**a**) Impact force and response; (**b**) Truncated transfer matrix (Note: ‘*’ is the mathematical convolution operation).

It is important to note that the group lasso regularization method is particularly time-consuming, since the transfer matrix is large in this inverse problem. In addition to the sparse feature of impact force, the nonzero force value in the specific time window could be located by analyzing structural dynamic responses, as illustrated in Figure 2a. For a subject subjected to multiple impacts, the total length of analysis time corresponds to the recorded length of the response. It should be noted that an impact typically occurs within a relatively small portion of the total analysis time. To improve the computational efficiency of reconstructing the impact force, an approximate impact window is used to reduce the sample sizes, and the corresponding portion of the transfer matrix is chosen for impact force identification. The impact window for truncation is determined by the excitation time and the duration of each impact force. The excitation time for each impact force is estimated from the dynamic responses, and the time duration is determined by the time window size. The time window delineated by a red rectangle could be used to reduce the columns of the transfer matrix into several variables, as illustrated in Figure 2b. This approach forms the basis of the proposed truncated transfer matrix-based *l*_2_-norm regularization (TML2) method, which offers an efficient and accurate solution to the problem:

(26) $$\underset{F_{ti}}{minmize}\;\;\;{\left\|H_{ti}F_{ti}-A\right\|}_{2}^{2}+\lambda {\left\|F_{ti}\right\|}_{2}^{2}$$

where $F_{ti}$ is the nonzero force vector at the location i, and $H_{ti}$ is the truncated transfer matrix.

#### 2.3.4. Summary

**Figure 3:**
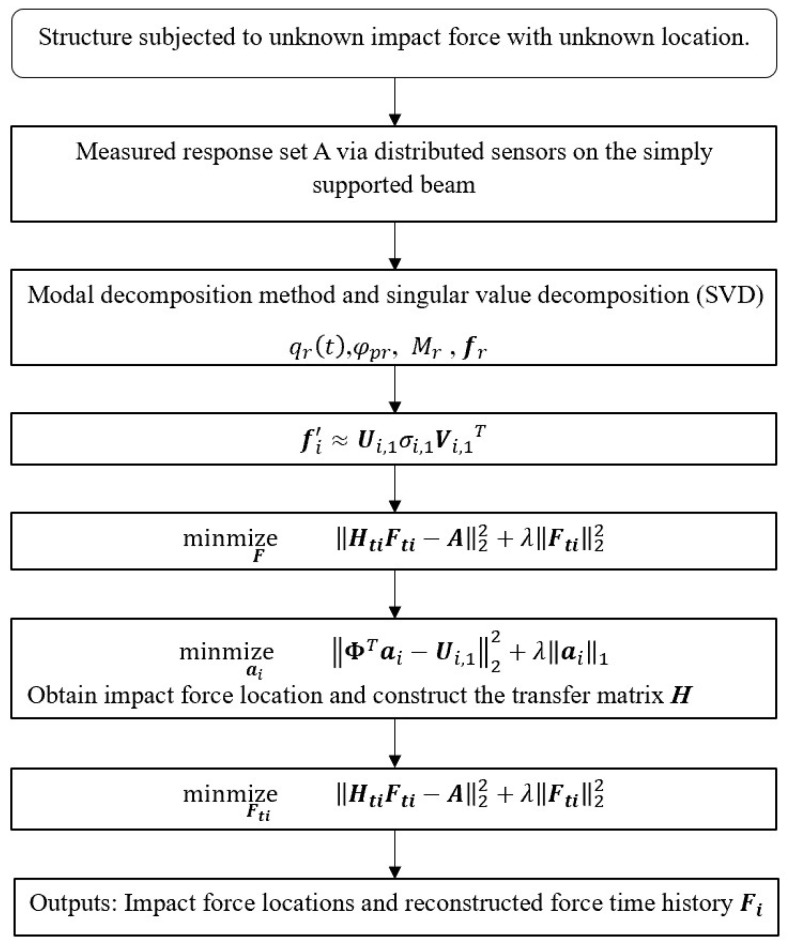
Flowchart of two-step impact force identification method.

The proposed method consists of two sequential steps. In the **first step**, the acceleration responses measured at multiple points are converted to modal coordinates using mass-normalized mode shapes obtained from an analytical or updated finite element model. The modal force vectors for each candidate load location are assembled into a matrix and decomposed via singular value decomposition (SVD). The dominant right singular vector reveals the pattern of the modal forces at that location. A sparse *l_1_*-norm regularization problem is then solved to obtain a coefficient vector for each candidate location; only a few nonzero coefficients remain at the true impact positions. In the **second step**, once the excitation positions have been determined, the transfer matrix is truncated to the short excitation window identified from the response, and a Tikhonov-type *l_2_*-norm regularization problem is solved to reconstruct the time history of the impact force. Truncation reduces column correlation and improves both accuracy and computational efficiency. The detail procedure of the proposed method is illustrated in Figure 3.

As shown in Figure 3, the algorithm requires selecting regularization parameters for both steps. For the sparse localization in the first step, the *l*_1_-regularization parameter is chosen from 50 logarithmically spaced candidates between 10^−4^ and 10^−1^ by minimizing the reconstruction error on synthetic data. This procedure is used throughout numerical and experimental studies. For the second step, the *l*_2_-regularization parameter is determined automatically using the generalized cross-validation (GCV) criterion. The sampling rate for all analyses is 1000 Hz. The impact force is assumed to act over a 0.01 s window, and the response window is extended to 10 s to include free-vibration decay.

## 3. Numerical Study

### 3.1. Numerical Modelling

**Figure 4:**
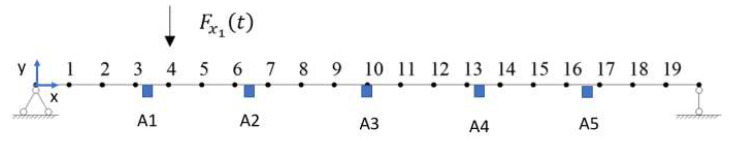
Numerical model of simply supported beam.

To evaluate the performance of the proposed method, a simply supported beam model is adopted. As shown in Figure 4, the beam model is considered as a one-dimensional structure with a length of 6 m and a cross-section of 0.1 m × 0.03 m with a mass density of 7850 kg/m^3^. The Young’s modulus *E* of the beam material is 2.0 × 10^11^ N/m^2^, and structural damping is considered as Rayleigh damping with two coefficients α = 0.5 and β = 1. The beam model is discretized into 300 equal Euler–Bernoulli finite elements. The first six natural frequencies of the beam are 1.95, 7.81, 17.5, 31.25, 48.83, and 70.32 Hz. The dynamic response of the beam is calculated with a time interval of 0.001 s and a measurement duration of 10 s to ensure that the entire impact excitation and free-vibration responses are captured. The impact force is represented as a triangular pulse, characterized by five nonzero values occurring within a 0.01 s time impact window. For the sparse regularization in the first step, the optimization problem is solved using an iterative shrinkage–thresholding algorithm. The regularization parameter λ is selected from 50 logarithmically spaced candidates in the range $\left[10^{-4},10^{-1}\right]$ based on the minimum reconstruction error on synthetic data. For the Tikhonov-type regularization in the second step, the generalized cross-validation (GCV) criterion is adopted to automatically determine λ.

Nineteen possible force locations, named $\left\{P_{1},P_{2},\ldots ,P_{19}\right\}$, are considered in force localization. These locations are uniformly distributed along the beam with an interval of 0.3 m. In the following, the number of sensors for acceleration measurements is five, seven, and nine, and sensor locations are evenly distributed along the beam. For example, for the case with five sensors, sensor locations are labelled as A1–A5 in Figure 4. Acceleration response measurements are used for impact force identification. A relative percentage error (RE) is defined to evaluate the identified accuracy of impact forces as

(27) $$RE=\frac{{\left\|f_{identified}-f_{true}\right\|}_{1}}{{\left\|f_{true}\right\|}_{1}}\times 100\%$$

where $f_{identified}$ and $f_{true}$ are the identified and true force vectors, respectively.

Moreover, a peak force relative percentage error (PRE) is defined to evaluate the identified accuracy of the peak impact forces as

(28) $$PRE=\frac{\left|max(f_{identified})\right|-|max(f_{true})|}{max(f_{true})}\times 100\%$$

White noise is added to simulate the measurement as

(29) $$A_{n}=A+lev\times \frac{1}{n}{\left\|A\right\|}_{1}\times rand$$

where $A_{n}\;and\;A$ are the measurements and calculated structural responses, respectively. n is the total number of elements in the vector A. lev is the noise level. ${\left\|A\right\|}_{1}$ is the *l*_1_-norm of the vector A. rand is a random vector with the normal distribution.

### 3.2. Single Impact Force Identification

**Figure 5:**
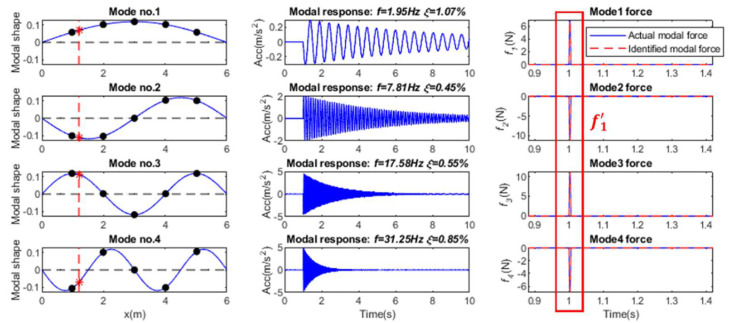
Modal parameters for single force identification (Note: ‘*’ is a mathematical convolution operation).

In this section, the identification of a single impact force at Position P_4_ is investigated. Based on the equally distributed acceleration responses and mass-normalized modal coefficient, the modal response under the impact force can be extracted, as shown in Figure 5. Then each modal force can be reconstructed from the modal response using the regularization method, and the force location can be determined by Equation (21). The effect of mode number, sensor number, and noise level on the identified results using different regularization methods is studied in this section.

#### 3.2.1. Comparison of Different Regularization Methods

**Figure 6:**
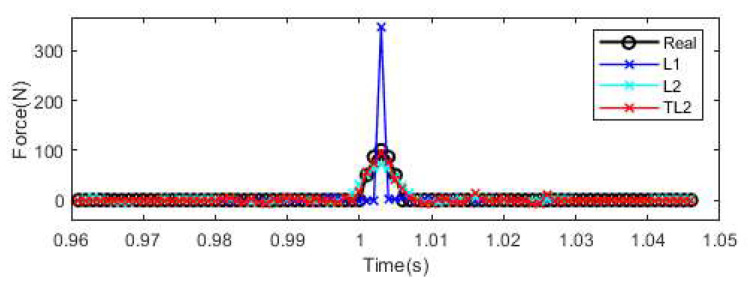
Identified impact forces with nine accelerometers with 10% noise level.

In this section, the performance of the *l*_1_-norm, *l*_2_-norm, and TML2 regularization methods in terms of force identification is studied. Nine equally distributed acceleration responses are selected. The first 10 modes are used to construct the transfer matrix. The 10% noise level is considered in this section. Table 1 shows the comparison results for Tikhonov regularization, standard sparse regularization, and truncated transfer matrix-based regularization for identifying the impact force. The reconstructed results of force value time history are shown in Figure 6.

From Figure 6 and Table 1, the relative errors in the identified impact force results from the Tikhonov (*l*_2_-form) and sparse (*l*_1_-form) regularization techniques are 30.45% and 142.54%, respectively, and their corresponding amplitude errors are 12.39% and 258.77%, respectively. These two errors from the proposed TML2 method are 6.41% and 1.86%, respectively. The *l*_2_-norm regularization method is very sensitive to measurement noise. Due to the high correlation existing inside the transfer matrix, the *l*_1_-norm regularization method is not suitable for impact force identification with five nonzero elements. Compared with existing methods, the accuracy of the proposed method is significantly increased. The computational time for these three methods is 24.73 s, 335.51 s, and 1.04 s, respectively. The results show that the proposed method has the highest computational efficiency. The proposed TML2 regularization method will be used for impact force identification in the following sections.

#### 3.2.2. Effect of Measurement Noise

**Figure 7:**
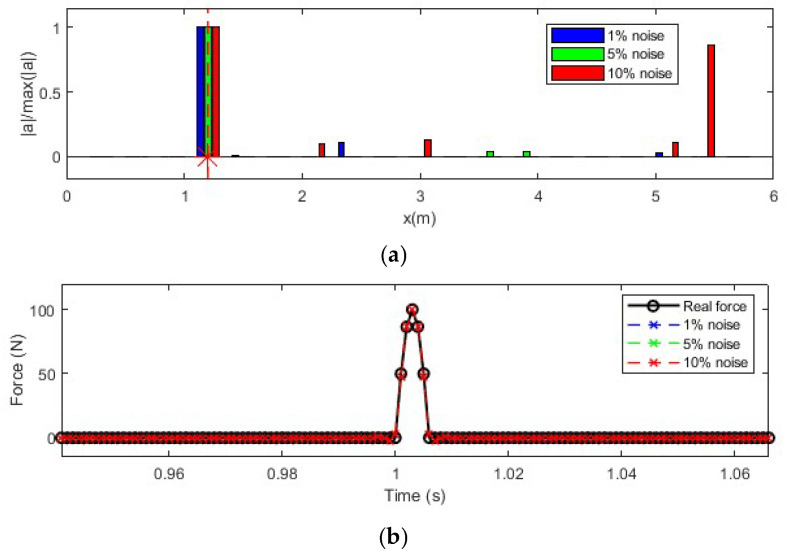
Effect of measurement noise on impact force identification. (**a**) Location identification (Note: ‘*’ is a mathematical convolution operation); (**b**) Force value identification.

In this section, the effect of measurement noise on the identified results is studied considering three noise levels, i.e., 1%, 5%, and 10%. Noise is added to simulate measurements. The identification results with the 1%, 5%, and 10% noise levels are shown in Figure 7. As shown in Figure 7a, there are variations in the identified force locations. When the noise level is 10%, there is a mis-identified force location around 5.5 m. To accurately locate the force position, signal processing techniques are needed to reduce measurement noise. As shown in Figure 7b, the identified force values from different measurement noises are very close. The results show that the proposed method is robust to measurement noise for force value identification.,

#### 3.2.3. Effect of Number of Modes

**Figure 8:**
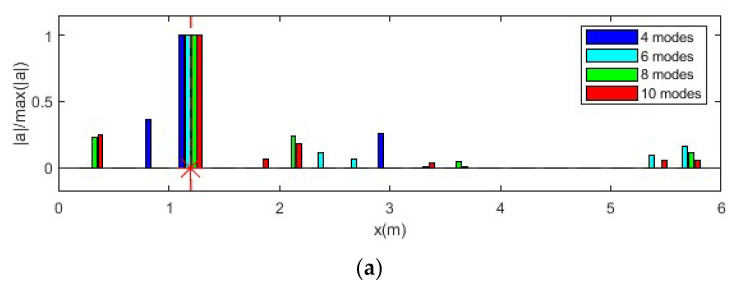

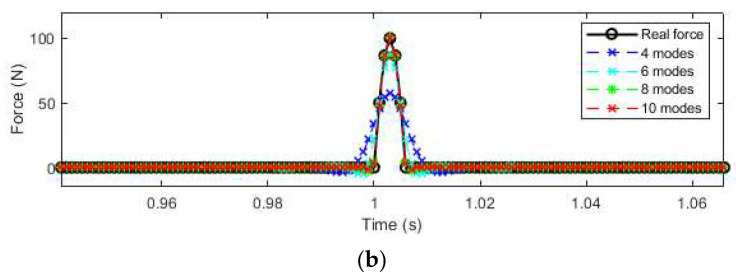
Force identification results with different numbers of modes. (**a**) Location identification (Note: ‘*’ is a mathematical convolution operation); (**b**) Force value identification.

In this section, the effect of the selected mode number on force identification is studied considering 5% measurement noise. A total of 4 modes, 6 modes, 8 modes, and 10 modes are selected for force location identification and force value reconstruction. Nine equally distributed accelerometers are selected. The reconstruction results are shown in Figure 8.

As shown in Figure 8a, the location of the impact force can be identified successfully using different numbers of modes. However, since measurement noise influences modal response extraction, especially for high-frequency modes, there are substantial errors in force location identification with a high number of modes considered. In Figure 8b, it is observed that the number of modes has a significant influence on force value identification. The identified result is much closer to the true value as the number of modes increases. This is because the transfer matrix is constructed based on the modal superposition method, and a transfer matrix with a low number of modes lacks high-frequency components. Consequently, the identified force value with fewer modes is much smaller than the true value, with the case using four modes being the most inaccurate. The results indicate that while increasing the number of modes improves the identification accuracy of the force value, it also introduces errors in force location identification due to the noise affecting high-frequency modes. Therefore, a balance must be struck between the number of modes used and the noise level to optimize identification accuracy.

#### 3.2.4. Effect of Number of Sensors

In this section, impact force identification using acceleration responses measured using different numbers of sensors is conducted. Three cases, i.e., five sensors, seven sensors, and nine sensors, are used. The 5% noise level is considered, and 10 modes are used for transfer matrix construction in this section. The identified results are shown in Figure 8.

**Figure 9:**
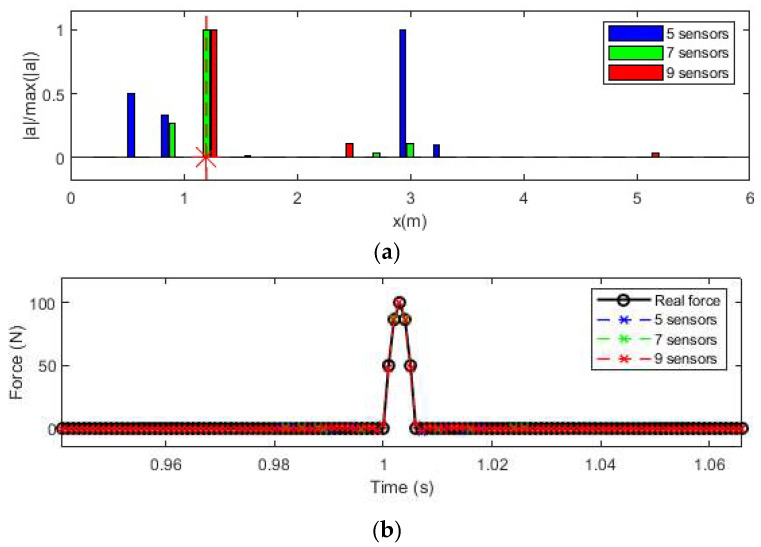
Force identification results with different numbers of sensors. (**a**) Location identification results (Note: ‘*’ is a mathematical convolution operation); (**b**) Force value identification results.

From Figure 9a, the location of the impact force is identified correctly when the number of sensors is seven or nine. When the number of sensors is five, the impact force location cannot be identified correctly. The results show that seven sensors are needed to identify the force location. With more sensors, a more accurate modal response could be decomposed from responses, and a more accurate load location coefficient could be extracted from the identified modal force. From Figure 9b, the identified results using five, seven, and nine sensors are approximately the same, and this shows that the number of sensors has no big influence on force value reconstruction. The RE and PRE of the identified force value compared to the true values are summarized in Table 2. From Table 2, the RE and PRE values are not larger than 1.82% even with the 10% measurement noise in the response. The results show that the proposed method is very robust to measurement noise. The results in this table also further confirm that the error of identified forces is reduced when the number of modes and sensors increases.

### 3.3. Multiple Impact Force Identification

**Figure 10:**
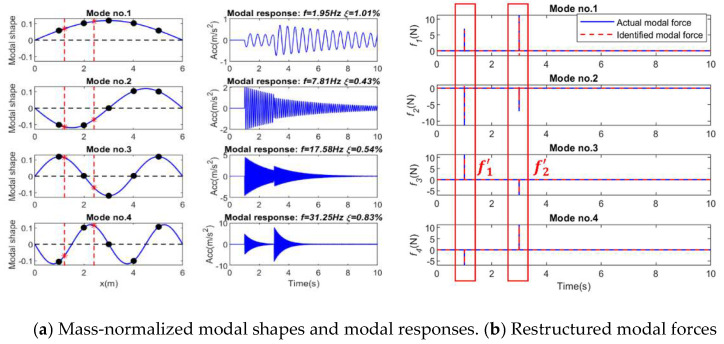
Modal parameters for identification of two forces (Note: ‘*’ is a mathematical convolution operation).

To verify the performance of multiple impact force identification, two impact forces are applied at Locations P4 and P8. Figure 10a shows the mass-normalized modal coefficient and the modal responses under these impact force excitations extracted from the acceleration measurements, respectively. Figure 10b shows the modal forces reconstructed from the modal response using the regularization method.

**Figure 11:**
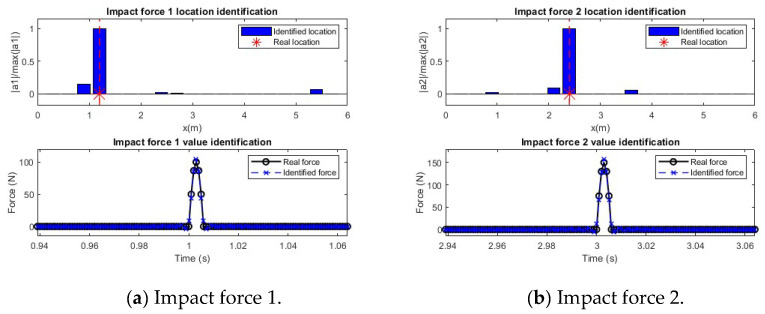
Identification and localization of two impact forces.

In this study, 10 modes are used for the transfer matrix. The number of sensors is nine, and the 5% noise level is considered in response measurements. Figure 11 shows the identification results of two forces. Figure 11a shows the identification and localization of the first impact force, and the results for the second impact force are shown in Figure 11b. From this figure, the locations of two impact forces are identified successfully by peaks, and the values at other locations are very small. The identified amplitudes of these two impact forces agree well with the true values. The results show that the proposed method can identify multiple impact forces accurately.

## 4. Experimental Validation

### 4.1. Experimental Setup

**Figure 12:**
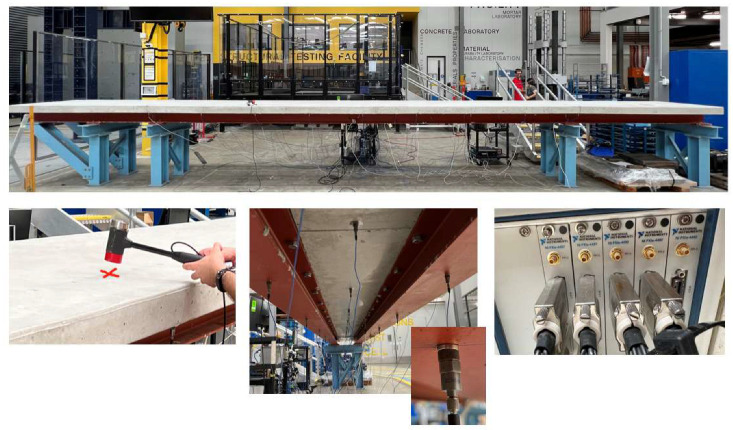
Experimental model and acquisition equipment.

**Figure 13:**
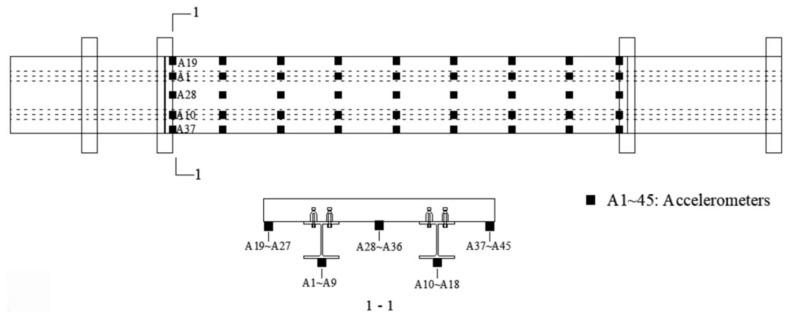
Arrangement of accelerometers.

To further verify the proposed method, a three-span steel–concrete composite beam bridge model (10,000 mm long, 1000 mm wide, and 300 mm thick) was built in the laboratory, as shown in Figure 12. The bridge spans are independent, and the left and right spans are 2000 mm. The middle span with a 6000 mm length was the main span for testing. The main bridge is a concrete slab on two steel I-beams connected by shear connectors. The concrete slab has a thickness of 100 mm and a width of 1000 mm. There are 45 accelerometers installed at the bottom of the bridge deck, and the arrangement of the accelerometers is shown in Figure 13.

### 4.2. Finite Element Model Validation

**Figure 14:**
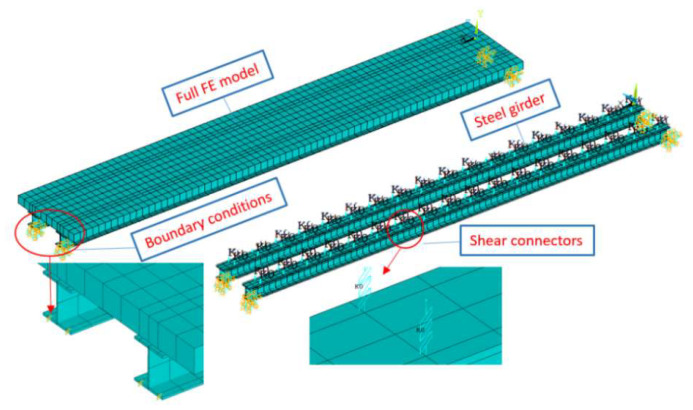
Finite element model.

The finite element (FE) model of the bridge was established using ANSYS 2021R2. A convergence study was conducted, and all components including the concrete slab, shear connectors, and steel beam were properly modelled, as shown in Figure 14. The concrete slab and the steel girders were modelled by the shell element with four nodes (Shell 63). The steel girder and concrete slab are connected via bolts as shear connectors, which are used to transmit the longitudinal shear force between the steel girders and concrete slab. A non-linear spring element (Combin39) was employed to model the shear connector. The positions of the spring elements coincide with the positions of the shear connectors in the composite beam. An elastic Young’s modulus of 205 GPa and Poisson’s ratio of 0.3 are defined for the steel girder. An elastic Young’s modulus of 30 GPa and Poisson’s ratio of 0.3 are defined for the concrete slab. The bottom of both ends of the steel girder are restricted to move in the X, Y, and Z directions to simulate the real boundary conditions, as shown in Figure 14. The effect of frictional contact between steel girders and the concrete slab is ignored in this model.

The mass-normalized mode shape coefficients can be calculated from the FE model for force identification. The errors of the FE model could affect the force identification results. The FE model was updated to ensure the best correlation between experimental and numerical frequency and mode shapes.

**Figure 15:**
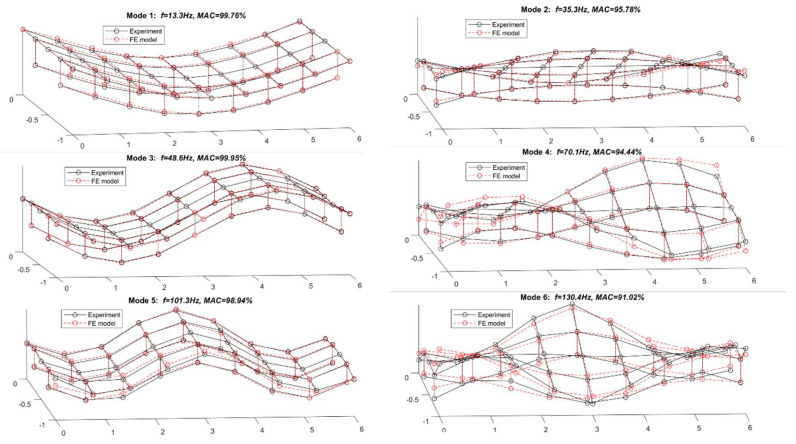
First 6 modal shapes from experimental and FE models.

A comparison between the experiment model and updated FE model regarding modal frequencies and mode shapes is presented in Table 3. Figure 15 shows the modal shapes of the first six modes from the experimental and FE models. From Table 3, the natural frequencies from experimental testing are very close to that from the FE model, and the maximum difference is 2.52% for the third torsional mode. The corresponding modal assurance criterion (MAC) values between the experimental and numerical mode shapes are all over 0.9100. Figure 15 further confirms that the modal shapes from the FE model agree well with that from experimental testing. The results show that the FE model is validated, and it could represent an experimental model. The validated FE model will be used to construct the modal shape coefficients in this study.

### 4.3. Impact Force Identification

**Figure 16:**
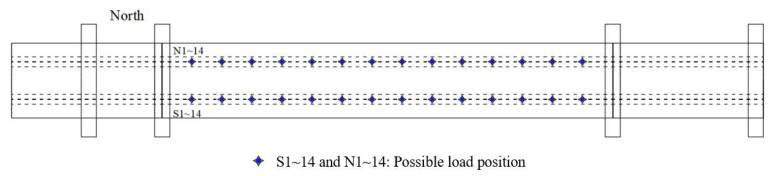
Predefined possible load positions in the experiment model.

In the experimental test, a total of 28 possible excitation locations are chosen in this study, and these locations are labelled as S1 to S14 and N1 to N14 in Figure 16. The acceleration responses from A1 to A18 are used for force identification. Single force identification and multiple force identification are conducted to verify the performance of the proposed method in this section.

#### 4.3.1. Single Force Identification

**Figure 17:**
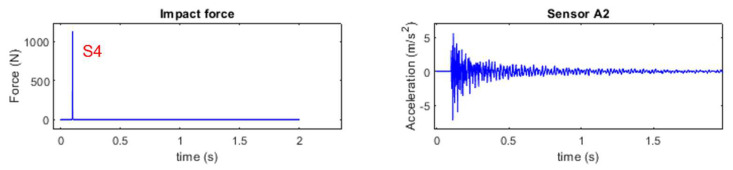
Time history of impact force at S4 and acceleration response from sensor A2.

For single force identification, hammer excitation is conducted on location S4, and the impact force and accelerations at A1 to A18 are captured. The signal sampling frequency is 1000 Hz. Figure 17 shows the impact force (located at S4) and the typical response time history at A2.

**Figure 18:**
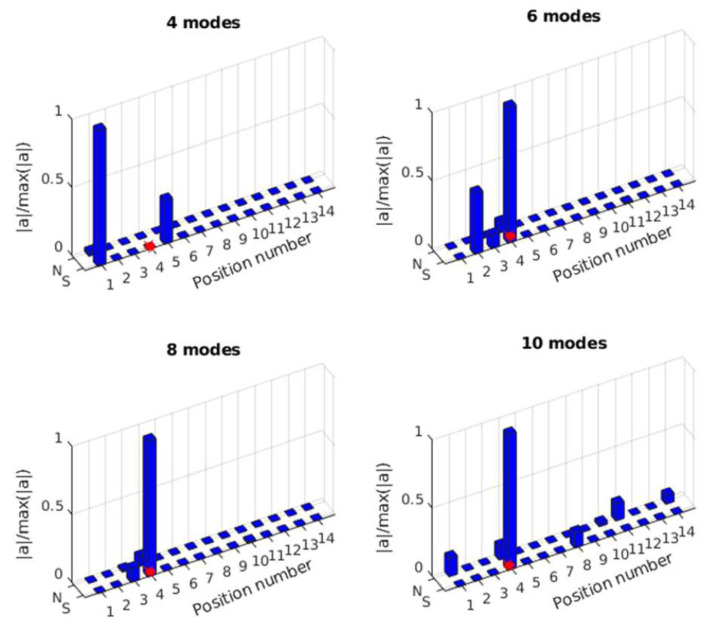
Identification of impact force at S4 with 4 modes, 6 modes, 8 modes, and 10 modes.

The mass-normalized modal shape coefficients are obtained from the validated FE model. Acceleration responses at A1 to A18 are used for force identification. From these acceleration responses, the modal responses are extracted. To reduce the effect of measurement noise, a bandpass filter is used to obtain the modal responses. Figure 18 shows the identified results of the impact force location using 4 modes, 6 modes, 8 modes, and 10 modes. From the results, the force location can be identified correctly when the number of modes is 6, 8, or 10. The force location cannot be identified correctly using four modes. This is due to the fact that force location information is embedded in the correlation coefficient of the modal force, and the accuracy of the modal force affects the identification of the force location. After obtaining the force location, the transfer matrix for force value identification can be constructed.

As shown in Section 3.2.3, the number of modes for constructing the transfer matrix has a large influence on impact force identification. In this experimental study, the frequency range of the measured acceleration response is up to 500 Hz as the sampling frequency is 1000 Hz. Only the limited modes in this frequency range are used to construct the transfer matrix. To reduce this effect, a lowpass filter is used, and the cut-off frequency is chosen to cover the modes to construct the transfer matrix. For comparison, 4 and 10 modes are used to construct the transfer matrix in this study.

**Figure 19:**
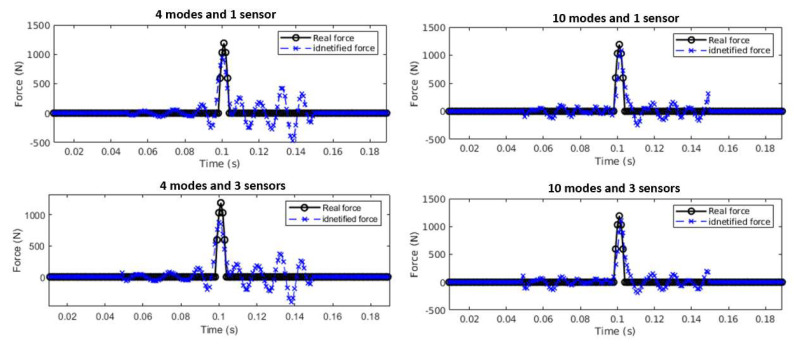
Single force identification with different numbers of modes and sensors.

The effect of the number of sensors is further verified using experimental measurements. Sensors are installed on the beam evenly to capture modal spatial information. Figure 19 shows the impact force identification results using different numbers of sensors and modes. The top two graphs show the results from one sensor at A2 using 4 and 10 modes. The bottom two graphs show the identified results from three sensors at A2, A4, and A6 using 4 and 10 modes. The results show that the force amplitude can be identified accurately for all cases. The result using 10 modes is better than that using 4 modes, and the results based on one and three sensors are approximately the same.

#### 4.3.2. Multiple Force Identification

**Figure 20:**
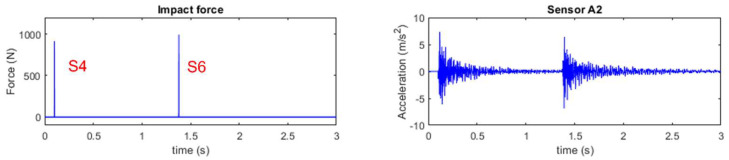
Multiple impact force identification from the acceleration response at A2.

For multiple force identification, hammer excitations are conducted on locations S4 and S6 separately. The signal sampling frequency is 1000 Hz. The accelerations of the bridge deck subjected to impacts are measured as the same as that in Section 4.3.1. Figure 20 shows the force (located at S4 and S6) and a typical response time history from A2.

**Figure 21:**
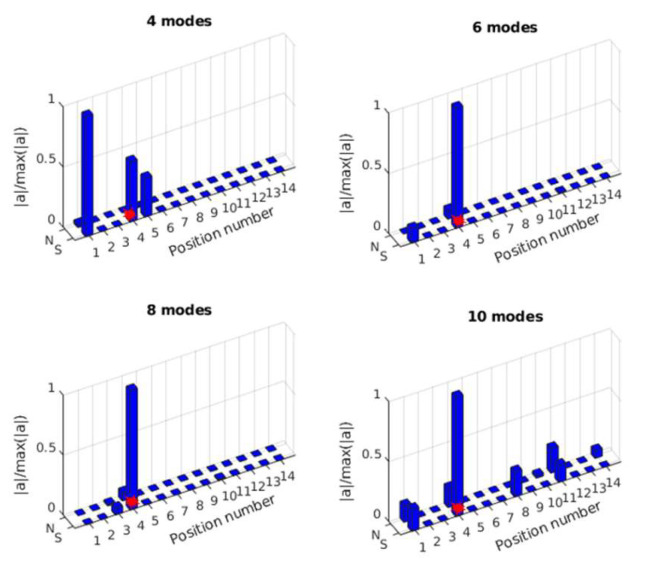
Identified impact force at S4 with 4, 6, 8, or 10 modes.

**Figure 22:**
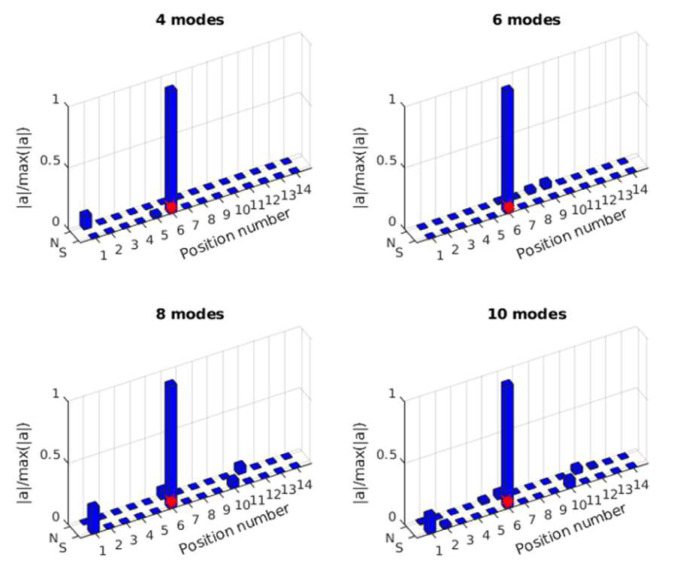
Identified impact force at S6 with 4 modes, 6 modes, 8 modes, and 10 modes.

A similar process for single force identification is implemented for multiple force identification. The mass-normalized modal shape coefficients are extracted using the validated FE model, and the modal responses are obtained from the measured acceleration responses of 18 sensors. As a comparison, 4, 6, 8, and 10 modes are used for force location identification. The results are shown in Figure 21 and Figure 22.

**Figure 23:**
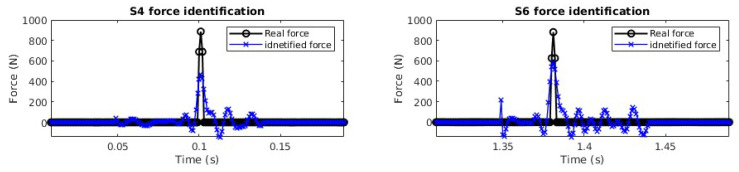
Identified impact forces at S4 and S6 with 10 modes and three sensors.

In Figure 21, the location of the impact force at S4 is identified successfully using 6, 8, and 10 modes, and there is an error using 4 modes. In Figure 22, the impact force at S6 can be correctly localized for all cases. After obtaining the force location, the transfer matrix for force value identification can be constructed. For multiple force value identification, 10 modes are used to construct the transfer matrix, and three sensors (A2, A4, A6) are used. Figure 23 shows the identified impact forces. The results show that two impact forces are identified successfully, and the amplitudes of the identified forces are a little smaller than the measured values.

### 4.4. Discussion

The proposed two-step method includes impact force localization and reconstruction, and the performance of the proposed method is verified on a beam model numerically and a bridge deck experimentally. The results are also compared with those from the classical Tikhonov *l*_2_-norm regularization method and standard sparse *l*_1_-norm method. The following observations were obtained:

(1) The numerical results show that the proposed method could accurately localize single and multiple impacts and reconstruct their time history with low computational cost. Increasing the number of modes improves localization accuracy, while the number of sensors primarily influences the reconstructed force amplitude. The proposed method is also very robust to measurement noise.
(2) The numerical and experimental results indicate that using the truncated transfer matrix with an impact time window could dramatically reduce column correlation and noise amplification. Sparse localization in modal coordinates provides reliable indicators of the true impact positions even when only a limited number of modes or sensors are available.
(3) A detailed comparison with existing regularization techniques is shown in Table 4. From this table, it is shown that the existing Tikhonov and sparse regularization methods need the pre-known impact excitation location and use the full transfer matrix. The Tikhonov regularization method provides stable solutions but tends to oversmooth sharp pulses, and it is sensitive to column correlation. Standard sparse *l*_1_-norm regularization could improve sparsity but still requires the force location a priori and underestimates peak amplitudes. Group sparse methods incorporate prior knowledge of the force group structure and have been shown to reduce relative errors, but they remain computationally expensive when considering many candidate locations.
(4) The proposed two-step method determines the location from the response data and applies a truncated transfer matrix for reconstruction, achieving high accuracy and efficiency. It could be extended to identity impact forces for large-scale practical structures.

**Table 4:** Analytical comparison of regularization strategies for impact force identification.

Methods	Assumptions	Advantages	Limitations
Tikhonov (*l*_2_)	Pre-known force location and full transfer matrix	Stable solutions, simple implementation	Oversmooth pulses; sensitive to noise and column correlation
Standard sparse (*l*_1_)	Pre-known force location and full transfer matrix	Sparse forces; reduces noise amplification	Underestimates peak amplitudes; requires location a priori
Proposed two-step method	Unknown location and truncated transfer matrix	Simultaneously localizes and reconstructs; high accuracy and computational efficiency; robust to noise	Requires modal parameters; two-stage implementation

## 5. Conclusions

In this paper, a novel two-step method was proposed for multiple impact force identification. The first step utilizes a sparse regularization method for impact force localization, and the second step employs a truncated transfer matrix-based regularization method to identify the impact force value. By leveraging the prior properties of the impact force, the transfer matrix could be truncated into specific features to eliminate the effect of unloading intervals. The modal parameters and the sparse feature are combined for precise force localization. The performance of the proposed method is verified numerically on a simply supported beam model and experimentally on a composite bridge model. The following conclusions can be obtained.

(1) The numerical results show that compared with the classical Tikhonov (*l*_2_-norm) and sparse (*l*_1_-norm) regularization methods, the proposed method demonstrates superior accuracy and time efficiency in impact force value identification. For single force identification with 5% measurement noise, the relative errors of the identified entire impact force and the identified amplitude based on the proposed method are 6.41% and 1.86%, respectively.
(2) The numerical and experimental results show that the proposed method is very robustness to measurement noise. Its localization accuracy increases with the number of modes, and there is no obvious effect of the number of sensors. The accuracy of the identified impact forces slightly increases with the number of sensors.
(3) Numerical simulations and laboratory experimental studies demonstrated the performance of the proposed method. Further study is needed to extend the proposed method to the impact force identification of practical complex structures. The adaptive parameter selection of the impact time window for the truncated transfer matrix also needs further study. In addition, integrating machine learning techniques with the proposed method will enhance the online identification of impact forces for practical complex civil infrastructures.

## Figures and Tables

**Table 1:** Identified errors using different regularization methods.

*l*_2_-Norm			*l*_1_-Norm			TML2		
RE (%)	PRE (%)	Time (s)	RE (%)	PRE (%)	Time (s)	RE (%)	PRE (%)	Time (s)
30.45	12.39	24.73	142.54	258.77	335.51	6.41	1.86	1.04

**Table 2:** Results of identification accuracy indicators RE and PRE.

	Noise Level			Number of Modes				Number of Sensors		
	1%	5%	10%	4	6	8	10	5	7	9
RE (%)	1.02	1.19	1.82	136.79	20.45	6.88	1.19	1.25	1.22	1.19
PRE (%)	1.02	1.19	1.82	39.98	12.86	2.34	1.18	1.18	1.17	1.17

**Table 3:** Comparison between experiment model and updated FE model.

Mode No.	Description	Modal Frequency (Hz)		Error	MAC
		Experiment	FE Model		
1	1st bending	13.3	13.44	1.05%	0.9976
2	1st torsion	35.3	34.92	−1.08%	0.9578
3	2nd bending	48.6	48.37	−0.47%	0.9995
4	2nd torsion	82.7	80.72	−2.39%	0.9444
5	3rd bending	101.3	99.63	−1.65%	0.9894
6	3rd torsion	130.4	127.11	−2.52%	0.9102

## Data Availability

Data are contained within the article.
